# How the Timing of Biological Processes Is Controlled and Modified at the Molecular and Cellular Level? 2.0

**DOI:** 10.3390/biology13030170

**Published:** 2024-03-07

**Authors:** Jacek Z. Kubiak, Małgorzata Kloc

**Affiliations:** 1 Dynamics and Mechanics of Epithelia Group, Institute of Genetics and Development of Rennes, UMR 6290 CNRS/University of Rennes, Faculty of Medicine, 2 Ave. du Prof. Léon Bernard, 35000 Rennes, France; 2 Laboratory of Molecular Oncology and Innovative Therapies, Department of Oncology, Military Institute of Medicine, 04-141 Warsaw, Poland; 3 Transplant Immunology, The Houston Methodist Research Institute, Houston, TX 77030, USA; 4 Department of Surgery, The Houston Methodist Hospital, Houston, TX 77030, USA; 5 Department of Genetics, MD Anderson Cancer Center, The University of Texas, Houston, TX 77030, USA

The correct timing of molecular and cellular events is critical for embryo development, cell/tissue homeostasis, and to functions in all organisms throughout their whole lives. Thus, it plays a major role in biology. Despite this obvious key role of timing in all biological processes, we do not know exactly how cells and organisms measure time and how they translate the information on time flow to the correct regulation of molecular processes.

A major obstacle to generating a complete understanding of time is that we do not have a satisfactory and exact definition of the notion of time and time flow. We are all aware of the notion of time flow, but we cannot say exactly what it is. Does time pass alongside us or inside us? Are we a part of time flow or only witnesses? These questions have troubled philosophers since the beginning of humankind, and we are far from reaching complete understanding [1].

We can only measure time passing. Despite great progress in dealing with time, we still do not know what we are measuring exactly. We measure the flow of time as if it were linear, unidirectional, and irreversible, but we do not have certitude that time really has these attributes or even that it exists outside of our psychological realm. Although physicists hold the key to time reality, for describing biological processes, we must adopt the existing and generally accepted parameters of time flow.

Cells and organisms also measure time flow, react to time passing, and may modify processes using time through accelerating, delaying, or postponing certain reactions. The Special Issue “How the Timing of Biological Processes Is Controlled and Modified at the Molecular and Cellular Levels 2.0” gathers nine articles (five original research articles and four review articles) dealing with this description. Here, we present a series of articles on selected aspects of temporal regulation in cells, tissues, and organisms. These articles contribute to our understanding of the role of time-dependent coordination between molecular pathways in physiological vs. pathological conditions.

Some of these articles present examples of the importance of the temporal regulation of cell cycle events. For example, the cell cycle must proceed in a well-defined time frame to assure that there is coordination between cell proliferation and an embryo genetical developmental program. Biochemical processes must progress using time with appropriate timing, and the correct sequence of events is strictly controlled using time to ensure their full coordination and purposefulness for the cell, tissue, and organism’s life. Checkpoint mechanisms monitor if the necessary processes have been completed before starting new ones. Thus, the precise timely coordination between molecular events/reactions/pathways and their specific regulation in different conditions allows for the harmonious functioning of cells, tissues, organs, and whole organisms.

Aadil Javed and colleagues [2] studied Hematological and Neurological Expressed 1 (HN1), which is the protein that has been previously shown to be especially highly expressed in prostate cancer cells. Its involvement in the cell cycle regulation was suggested due to a very specific pattern of expression: it is inversely correlated with cyclin B1 (the regulator of the major cell cycle kinase CDK1 or p34^cdc2^). They show that HN1 interacts with Cdh1 (a co-factor of APC/C involved in cyclin B degradation, and thus controlling CDK1 activity). HN1 appears as a novel cell cycle-regulated protein with potentially dual roles: the modulation of cell cycle progression at the S-phase and mitotic exit. It is potentially involved in the timely and regulated fluctuations of CDK1 activity.

Florian Pontheaux et al. [3] describe studies on the regulation of the maternal mRNA involved in the timely regulation of cleavage divisions occurring during the first steps of the sea urchin *Paracentrotus lividus’s* embryo development. They show that IF4B orthologs present four specific domains essential for eIF4B function, and they copurify with eIF4E in a heterologous system. The injection of a morpholino directed against eIF4B mRNA downregulates the translational activity and delays cell divisions in embryos of two different sea urchin species. Conversely, the injection of an mRNA encoding for *P. lividus* eIF4B increases translation and accelerates the cleavage rate in embryos of the two species. Their data suggest that eIF4B mRNA *de novo* translation takes part in a conserved regulatory mechanism contributing to the regulation of both the level of protein synthesis and the cell division rhythm during early sea urchin development.

The review article by El Dika and colleagues [4] summarizes how the timing of mitotic divisions is tuned by a mechanism dependent on CDC6 ATPase, which inhibits the major mitotic kinase CDK1 (or p34^cdc2^). CDC6 was well known as a regulator of the S-phase of the cell cycle. It appears that it also plays a critical role in the timely regulation of the M-phase. In *Xenopus laevis* embryos, CDC6 brings a *bona fide* CDK1 inhibitor Xic1 into proximity with CDK1 and inhibits this kinase. This inhibition allows for good regulation of both the timing of the mitotic entry and the duration of the M-phase. CDC6 is, therefore, an important regulator of the timing of embryonic cleavage divisions allowing for coordination between the cell cycle events and the genetical program of embryo development.

Another review article by Avraham Greenberg and Itamar Simon [5] presents methodologies to measure the S phase duration of the cell cycle. The authors summarize the current knowledge of the remarkable, and often ignored, variability of the duration of this essential cell cycle phase. They focus on the organization of the DNA replication program and the degree of overlap between the firing of replication domains, which are the main determinants of the S phase duration, and how it impacts the whole cell cycle duration.

The article by Courtney Leung and colleagues [6] provides the link between the abovementioned cell cycle studies and those devoted to chronobiology and circadian rhythms. The authors use a computational model of a molecular network determining the succession of cell cycle phases and, via numerical simulations, describe the consequences of the circadian control on these events. For instance, this allows for the prediction of optimal timing for anti-cancer-drug administration targeting effectively selected phases of the cell cycle on which the drugs act most efficiently. This approach is the basis for the elaboration of chronopharmacological protocols in the treatment of different diseases including cancers.

The review article by Yool Lee and Jonathan P. Wisor [7] discusses how circadian clocks reciprocally interact with other signaling and metabolic factors to coordinate daily rhythms. Numerous cellular and animal models demonstrate the presence of functional circadian oscillators at multiple levels, ranging from individual cells, like neurons or fibroblasts, to the brain and peripheral organs. These oscillators are tightly coupled to the timely modulation of cellular and bodily responses to physiological and metabolic cues. The authors focus on the roles of central and peripheral clocks in physiology and diseases, and their relationship to regulatory interactions between circadian timing systems and molecular and metabolic factors.

The article by Viacheslav V. Krylov and collaborators [8] focuses on the relationship between daily changes in illumination and diurnal geomagnetic variations at the organismal level using the zebrafish *Danio rerio* as an experimental model. They analyzed circadian patterns of the locomotor activity of zebrafish under different combinations of light regimes and slow magnetic fluctuations, based on a record of natural geomagnetic variations. The results show that slow magnetic fluctuations can induce endogenous rhythmic activity and point to the photosensory receptors, the cryptochromes, as the potential key molecular actors in these processes.

Cycling events take place on yet another level in the human endometrium, playing a key role in establishing pregnancy. Mladen Naydenov et al. [9] show that microRNAs play essential functions in human endometrium development during the menstrual cycle. They demonstrate that miR-449a/c and their sequence variants (isomiRs) participate in the genetic control of human endometrial receptivity. Moreover, they found that the expression of miR-449c.1 and its precursor (pre-miR-449c) correlated with the histological assessment of the endometrial phase and patient age. This study may instigate clinical investigations and potential applications of the miR-449 family in the diagnosis and prognosis of human reproductive diseases.

The review article by Kloc et al. [10] defines senescence and aging and describes how organismal senescence is determined by molecular and cellular senescence. They discuss the cellular and molecular features of senescent cells and the role of multinucleated giant cells in aging organs. They also describe how senescence reversal and multinucleated giant cells initiate cancer and lead to cancer progression and metastasis. Although it was always believed that senescence is irreversible, many current studies indicate that in various types of cancer, the senescent multinucleated giant cells overcome senescence in various cancers producing highly aggressive mononucleated stem-like cells, which divide and initiate tumor development and progression.

WHO estimates that the population aged 60 years and over will increase from 1 billion in 2020 to 1.4 billion by 2030, and 2.1 billion by 2050. Additionally, the number of people aged 80 years or older will triple between 2020 and 2050 to a total of 426 million. This rapidly expanding aging population and extension of human life is another reason why, besides scientific curiosity, a full understanding of time-related molecular and cellular processes is especially relevant in the modern world, and it provides great hope for the development of future clinical therapies for age-related diseases.
